# An artificial intelligence lightweight blockchain security model for security and privacy in IIoT systems

**DOI:** 10.1186/s13677-023-00412-y

**Published:** 2023-03-16

**Authors:** Shitharth Selvarajan, Gautam Srivastava, Alaa O. Khadidos, Adil O. Khadidos, Mohamed Baza, Ali Alshehri, Jerry Chun-Wei Lin

**Affiliations:** 1 Department of Computer Science, Kebri Dehar University, Kebri Dehar, Ethiopia; 2 Department of Math and Computer Science, Brandon University, R7A 6A9 Brandon, Canada; 3 Research Centre for Interneural Computing, China Medical University, 40402 Taichung, Taiwan; 4 Dept. of Computer Science and Math, Lebanese American University, 1102 Beirut, Lebanon; 5 Department of Information Systems, Faculty of Computing and Information Technology, King Abdulaziz University, Jeddah, Saudi Arabia; 6 Department of Information Technology, Faculty of Computing and Information Technology, King Abdulaziz University, Jeddah, Saudi Arabia; 7 Department of Computer Science, College of Charleston, Charleston, USA; 8 Department of Computer Science, University of Tabuk, Tabuk, Saudi Arabia; 9 Department of Computer Science, Electrical Engineering and Mathematical Sciences, Western Norway University of Applied Sciences, Bergen, Norway

**Keywords:** Artificial intelligence, Blockchain, Convivial Optimized Sprinter Neural Network, Cloud computing, Fog computing, Security

## Abstract

The Industrial Internet of Things (IIoT) promises to deliver innovative business models across multiple domains by providing ubiquitous connectivity, intelligent data, predictive analytics, and decision-making systems for improved market performance. However, traditional IIoT architectures are highly susceptible to many security vulnerabilities and network intrusions, which bring challenges such as lack of privacy, integrity, trust, and centralization. This research aims to implement an Artificial Intelligence-based Lightweight Blockchain Security Model (AILBSM) to ensure privacy and security of IIoT systems. This novel model is meant to address issues that can occur with security and privacy when dealing with Cloud-based IIoT systems that handle data in the Cloud or on the Edge of Networks (on-device). The novel contribution of this paper is that it combines the advantages of both lightweight blockchain and Convivial Optimized Sprinter Neural Network (COSNN) based AI mechanisms with simplified and improved security operations. Here, the significant impact of attacks is reduced by transforming features into encoded data using an Authentic Intrinsic Analysis (AIA) model. Extensive experiments are conducted to validate this system using various attack datasets. In addition, the results of privacy protection and AI mechanisms are evaluated separately and compared using various indicators. By using the proposed AILBSM framework, the execution time is minimized to 0.6 seconds, the overall classification accuracy is improved to 99.8%, and detection performance is increased to 99.7%. Due to the inclusion of auto-encoder based transformation and blockchain authentication, the anomaly detection performance of the proposed model is highly improved, when compared to other techniques.

## Introduction

The Industrial Internet of Things (IIoT) [1, 2] is becoming increasingly recognized as a potential component for re-designing existing industries. It accomplishes this by offering enormous benefits to manufacturing units, such as information gathering, advanced analytics, and monitoring of entire systems. IIoT [3] is an advanced version of IoT and is used in many application systems for smart cities, smart homes, security, and health monitoring, often operating on Cloud-based infrastructure and on the Edge of Networks. IIoT combines a collection of sensors with smart devices to determine the status of industrial machines, collect data for large application systems, and enable massive data transmission [4–6]. IIoT combines the advantages of automation technologies with reliable machine-to-machine communication, improvement in managing large-dimensional data, and high learning capability. In this environment, monitoring, big data collection, and information analysis are mainly performed using communication and interface devices (edge of networks) with centralized storage on the Cloud [7–10]. Data can then be uploaded to Cloud based systems on a regular basis using intermediate servers or gateways in a given area [11]. However, this communication infrastructure is more vulnerable to security breaches, as an untrusted cloud server may want to access a significant amount of sensitive data. Therefore, it is even more important to provide these systems with security [12–15] to ensure the privacy and confidentiality of industrial data. To this end, traditional work has developed blockchain technology, which is a distributed ledger-based cryptographic mechanism [16, 17]. It is typically used to store timestamped information from transactions in data blocks that are linked together to construct a chain using the chronological order of transactions [10, 18, 19]. Each data block has a specific hash value that is generated using a cryptographic method to ensure integrity of the data. These hash values link these blocks together like a linked list. Similarly, Artificial Intelligence (AI) [20–23] has become one of the most popular technologies used to ensure the security of IIoT systems.

The addition of numerous industrial sensors allows conventional Cyber Physical Systems (CPS) to maintain data availability. However, these methods are inadequate given the possibility of faulty and compromised sensors. In IIoT, these compromised sensors may send inaccurate data. There needs to be some infrastructure that can be trusted where these problems can be seen in order to distinguish between unreliable and reliable sensors (which sensors are faulty and which are reliable). Therefore, maintaining trust among IIoT sensors is a crucial component in the development of a secure CPS. In the field of CPS, a number of methods and techniques have been put forth for preserving confidentiality, security, and trust and for the detection and prevention of cyberattacks. However, some of the issues that must be resolved for the development of CPS in an IIoT environment are not covered by these techniques. The development of a method that protects users’ privacy while transforming original data in such a way that personal information is kept confidential even after data mining processes have completed is a difficult task [24, 25]. Thus, the proposed work intends to develop a new framework using blockchain based privacy preservation and anomaly detection mechanisms.

The originality of the proposed work is, it incorporates the advantages of lightweight blockchain technology and machine learning mechanisms with simplified and enhanced security operations. The key contributions of this research work are as follows:

- Develop a security model for IIoT systems, an Artificial Intelligence-based Lightweight Blockchain Security Model (AILBSM) is proposed that combines the advantages of blockchain for privacy preservation and AI for attack classification.
- Ensure the privacy of IIoT systems with minimal computational complexity, a Lightweight Consensus Proof-of-Work (LCPoW) based privacy preservation mechanism is used.
- Reduce the impact of attacks by transforming features into encoded data, an Authentic Intrinsic Analysis (AIA) model is used to help improve the overall performance of attack detection.
- A novel Convivial Optimized Sprinter Neural Network (COSNN) algorithm is implemented to predict and classify attacks based on the features obtained from privacy preservation modules.
- Validate and test the performance of the proposed AILBSM framework through extensive experimental analysis to separately evaluate and compare the results of blockchain and AI mechanisms based on various parameters.

The remainder of this paper are divided into the following Sections: The next section summarizes the related works. The Research methodology section provides a detailed explanation of the proposed AILBSM-based security framework with the corresponding block and mathematical illustrations. The next section presents the results and discussion analysis of the proposed privacy preservation and AI-based attack detection mechanisms based on various parameters. Finally, the whole paper is summarized with the results and future scope in the Conclusion.

## Related works

This section investigates some of the baseline machine learning/deep learning based blockchain models used for securing IIoT systems. It also investigates the pros and cons of existing works according to security performance and outcomes.

Duraisamy et al. [24] implemented Krill-Herd (KH) optimization with an integrated Deep Learning Neural Network (DLNN) technique to improve the security of smart city networks. KH optimization is one of the most popular optimization techniques which is widely used for feature selection and dimensionality reduction. In addition, the min-max normalization mechanism was used to pre-process the given dataset. The main advantages of this work were higher recognition accuracy, high level of security and minimal time consumption. However, there are also limitations such as difficulty to implemen complex system model, lower convergence rate, and intricate mathematical calculations. Alsarhan et al. [25] employ a Support Vector Machine (SVM) classification technique for intrusion detection in Vehicular Ad-hoc Networks (VANETs). Three different types of optimization techniques, such as Particle Swarm Optimization (PSO), Ant Colony Optimization (ACO), and Genetic Algorithm (GA) were used separately to select the most suitable technique. In this work, it was shown that the combination of GA-SVM outperforms other approaches with better performance results. In addition, GA-SVM offers the key advantages of a lower number of false positives, a lower error rate, and a higher convergence speed. Bangui et al. [26] presented a comprehensive review of the latest machine learning techniques used to develop an advanced Intrusion Detection System (IDS). The IDS includes the widely used recurrent neural network (RNN), game theory, SVM, K-means, self-organizing map (SOM), logistic regression (LR), and random forest (RF) mechanisms. Among other mechanisms, RNN provides higher detection accuracy and efficiency. Maseleno et al. [27] deployed a Random Monarch Butterfly (RMB) optimization with integrated RNN technique to protect smart society networks from cyber threats. During the optimization, the migration and butterfly adaptation operators were used to identify the best optimal solution with a reduced number of iterations. Moreover, the attack detection performance of this system was validated and tested using the parameters of detection level, F-measure, accuracy, and error rate. The main advantages of this technique were the ability to handle large dimensional datasets and reduced training and testing time.

Matthew et al. [28] implemented a collaborative IDS framework for enabling secured data transactions in IIoT systems. The purpose of this work was to implement a blockchain based IDS framework for spotting cyber attacks in IIoT systems. Rathee et al. [29] utilized a Viterbi algorithm for implementing a blockchain based IDS framework to ensure security in IIoT. Moreover, their framework recognizes anomalies and/or intrusions in the network with reduced false positives and increased accuracy. Hewa et al. [30] designed a new security architecture based on blockchain and fog computing for increasing security of IIoT-cloud networks. Also, this work reduced network latency, and the central point of failure by integrating the cloud with IIoT systems. Table 1 presents a survey on existing literature with pros and cons.

**Table 1:** Survey on the existing literature works

Methods	Description	Pros & Cons
Fuzzy Keyword Search [2]	It aims to retrieve the EMR based on the fuzzy keyword search securely.	Lack of reliability, and computational burden.
Machine learning based IDS [7]	Here, a lightweight IDS framework is developed for protecting Edge IIoT systems from intrusions.	Efficient in data handling, easy to understand, and high time consumption.
Shamir’s threshold cryptography [8]	A blockchain-based cryptographic model is implemented to assure the data privacy in IIoT systems.	Ineffective decision making, high time for encryption and decryption.
KH-DLNN [24]	It uses the optimization integrated deep learning model for categorising the attacking events to ensure security.	Reduced time consumption for classification and slow convergence.
GA- SVM [25]	It predicts the intrusions according to the features of the dataset provided by GA.	Not suitable for large-scale applications, overfitting, and overlapping.

Most of the existing research has focused on developing blockchain technology to secure cloud and Fog-based systems. However, the literature has significant drawbacks [31–38] like high computational complexity, higher time consumption for classifier training and testing operations, and increased false predictions. Due to the complex computational operations, some of the existing approaches are difficult to deploy. The addition of numerous industrial sensors allows conventional CPS to maintain data availability. Traditional security approaches have several drawbacks and are inappropriate for IIoT systems, such as the ability of secure end-to-end encryption to impede analytical processes and raise false alarm rates. Hence, the proposed work intends to develop a new security model by using a blockchain based AI model for IIoT systems. The novel concept of the proposed framework is that it uses blockchain based privacy preservation and AI based attack detection for securing IIoT systems through BlockFog and BlockCloud platforms. Moreover, reputation based trust management is also implemented to ensure valid transactions in the network. This framework builds a trust monitoring system using an addressed-based blockchain reputation system to ensure that data produced by IIoT sensor devices is not tampered with or mis-represented. Two levels of privacy-preserving approaches are described in order to preserve privacy in IIoT-driven CPS. For data authentication and attack prevention, the initial level of blockchain services uses the Lightweight Consensus Proof-of-Work (LCPoW) algorithm. The second level employs a Convivial Optimized Sprinter Neural Network (COSNN) approach to encode features in order to counter an AI-learnable inference attack.

## Research methodology

**Fig. 1:**
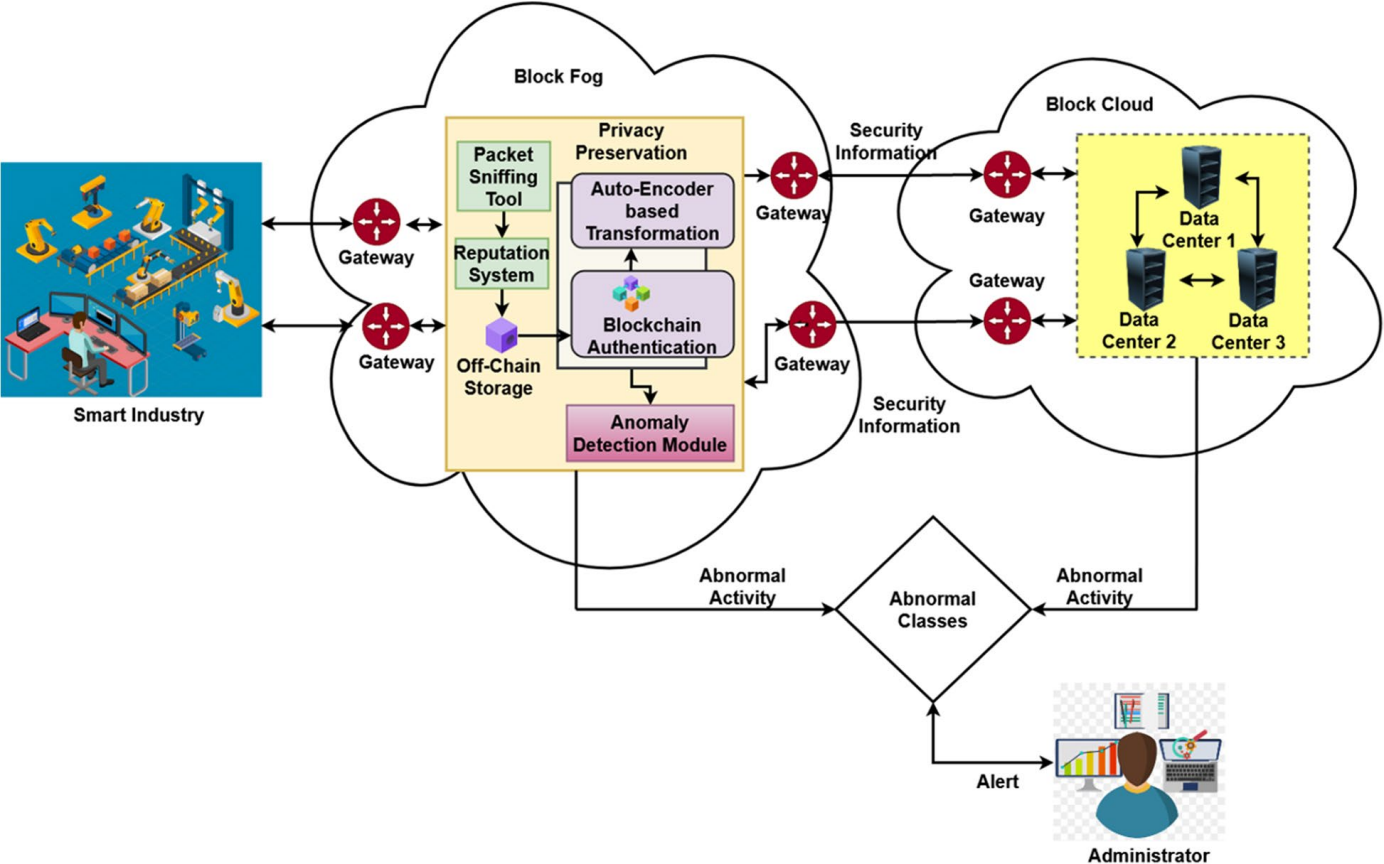
Working architecture model of the proposed AILBSM

The proposed Artificial Intelligence based Lightweight Blockchain Security Model (AILBSM) incorporates the mechanisms of trust management, blockchain-based privacy preservation, and AI-based attack detection. The general working model of the proposed AILBSM framework is shown in Fig. 1. It includes three levels of security operations: trust evaluation to verify the trustworthiness of the IIoT sensor device, data authentication and attack prevention using a lightweight blockchain algorithm, and attack classification using an AI mechanism. Trust verification validates data generated by IIoT sensor devices to verify whether a machine is tampered with or misdirected. It also uses a lightweight consensus Proof-of-Work (LCPoW) algorithm to ensure the privacy preservation of IIoT systems. Then, it performs data authentication to protect the IIoT system from dangerous attacks/intrusions. Moreover, an Authentic Intrinsic Analysis (AIA) mechanism is used to reduce the impact of attacks by converting the features into encoded data. Finally, the novel Convivial Optimized Sprinter Neural Network (COSNN) algorithm is used to accurately classify normal and intruder data based on the input data received from the privacy preservation module. Some of the most popular and publicly available cyber attack datasets were used to validate and test this framework.

### Trust management

Current CPS are more vulnerable to a wide variety of threats, including advanced adaptive threat attacks on physical layer connections, which can have physically devastating effects. Also, CPS are susceptible to both physical and digital forms of attack, the two most common of which are the former and the latter respectively. Cyber attacks are carried out by using malicious software such as malware or ransomware, or by accessing various components of network systems. In contrast, physical attacks involve the manipulation and exploitation of physical components. In contrast to active attacks, which have the potential to change data using inference attacks or data poisoning attacks, passive attacks involve hackers sniffing data from the CPS using publicly available data. Attackers will attempt to change typical data in order to commit a data poisoning attack. False data injection attacks are among the most common types of data poisoning attacks that can be launched against CPS networks. Therefore, one of the most important requirement of CPS is to make certain that data integrity as well as their safety is protected. The proposed privacy-preserving architecture includes three layers: application layer, network layer, and device layer. First, the device layer can generate data observed by the IIoT sensing devices. Then, their authenticity [39] is validated in parallel to ensure data security.

- ***Reputation-based Trust Estimation (See Algorithm 1)*** In the reputation based trust estimation model, parameters such as trust score, threshold value, and transaction value are initialized first. Then, the reputation score is estimated according to the number of transactions, which is further categorized based on the computed trust score value and total number of extracted features. Based on that, valid, reliable, and malevolent transactions are categorized.

**Figure:**
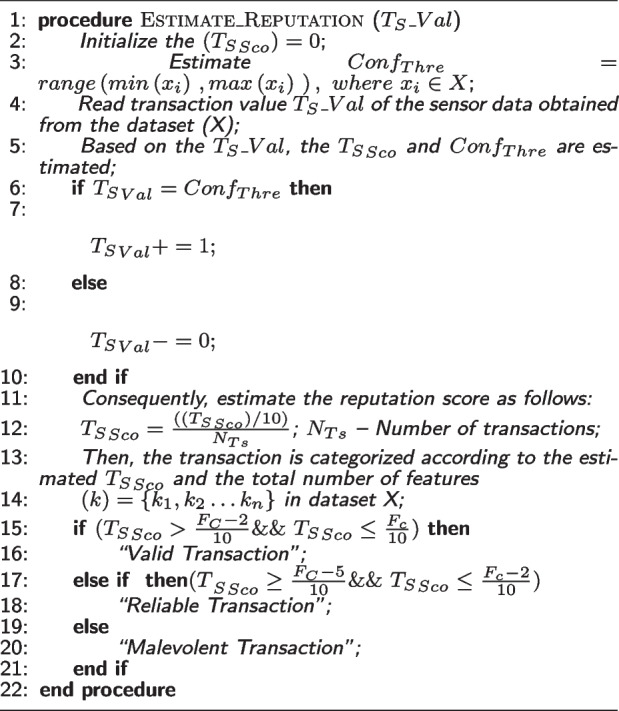
**Algorithm 1** Reputation-based Trust Estimation

Typically, data obtained from the physical environment may contain noise and manipulated information because a malicious entity exists. Therefore, it is important to ensure trustworthiness of the blockchain framework to guarantee security and privacy requirements.

During the off-chain data storage operation, transaction sensor information is obtained as output and the addressable hash value is produced as output. Consequently, parameters such as number of transactions and addressed hash value are initialized to 0. Moreover, for all sensor device in the network, the transaction value is obtained from each sensor device. If it is valid, the content addressed hash value is estimated and stored into the InterPlanetary File System (IPFS) of the server. After that, sensor devices store the information in the distributed hash table, and the addressed hash value is further transmitted to the BlockCloud and BlockFog platforms for future data access operations.

**Table 2:** List of symbols and descriptions

Symbols	Descriptions
$PF=\{PF_1,PF_2...PF_N\}$	Set of fog nodes
$PF=\{IS_1,IS_2...IS_n\}$	Sensor nodes
$T_{s_{Sco}}$	Trust score
$Conf_{Thre}$	Confident threshold
$min(x_i),max(x_i)$	Minimum and maximum values of the data
$T_{s_{Val}}$	Transaction value
$N_{Ts}$	Number of transactions
$(k)=\{k_1,k_2,\ldots , k_n\}$	Total number of features
*X*	Dataset
$C_{Adr}$	Content addressed hash value
$Tr_N$	Number of transactions
$Dis_H$	Distributed hash table
$Tr_N$	Number of transactions
$Pre_H$	Previous hash value
$Blk_{idx}$	Block index
*t*	Timestamp
*p*	Proof
$Cu_H$	Current hash value
$r_1 and r_2$	Random parameters
$h_i and q_i$	Data points for the given attributes
$Cr_e$	Correlation efficient
$k_i$	Data sample
$c_i$	Target class
*M*	Orthogonal matrix
*G*	Matrix column
$\epsilon _i$	Eigen value

**Fig. 2:**
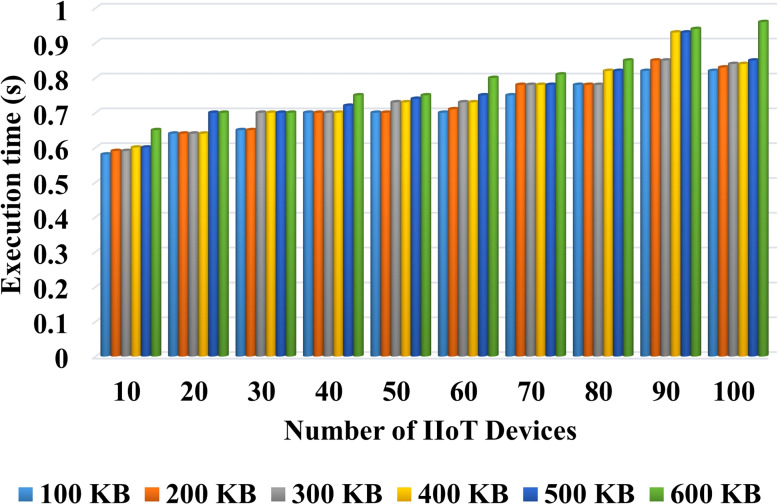
Execution time Vs No of IIoT devices

To this end, the proposed AILBSM framework employs a reputation-based trust estimation model to facilitate trust management in IIoT systems. Then, the network layer includes the set of fog nodes $BF=\{{BF}_1, {BF}_2, \dots , {BF}_N\}$ to formulate the BlockFog (BF) architecture, where each node correlates with the others based on peer-to-peer mode with sensor nodes $IS = \{{IS}_1, {IS}_2, \dots , {IS}_n\}$. Then, as shown in Fig. 2, the blocks in the BF and BC environments can be generated and released based on the confidence value. The consolidation of symbols and tables are presented in Table 2. Here, the trust value is estimated based on the trust score of the transaction and each transaction is compared with the minimum and maximum values of the confidence threshold in the dataset. Then, sensor devices with higher trust values are treated as trusted devices. Based on this process, normal, good, and malicious transactions are categorized in the IIoT systems.

- ***Off-Chain Data Storage (See Algorithm 2)***

**Figure:**
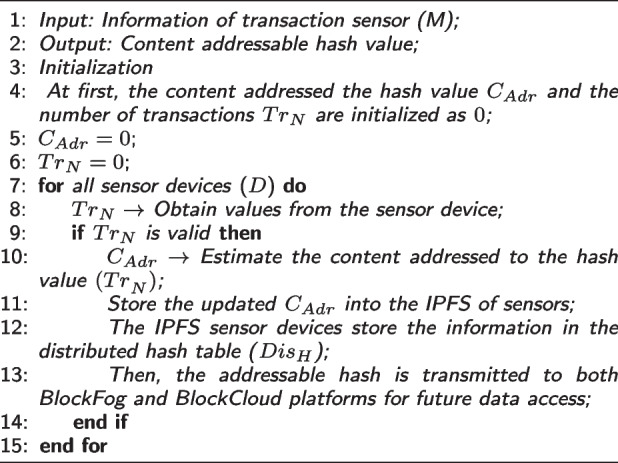
**Algorithm 2** Off-chain data storage

In this phase, the storage of data is done outside the chain to store information in the IPFS according to the category of transactions. Here, mainly reputation is estimated to determine the trustworthiness of the sensor device in IIoT systems. If the identified transaction is valid, the information collected from IIoT sensors will be stored appropriately in the IPFS, ensuring security and privacy. It also supports data duplication prevention by generating a unique addressable hash value.

### Blockchain based privacy preservation

In this work, two levels of privacy preservation mechanisms are used to ensure security of IIoT systems. At the first level, a highly efficient blockchain-based Lightweight Consensus Proof-of-Work (LCPoW) algorithm is implemented to authenticate data to protect IIoT systems from harmful attacks. Consequently, an Authentic Intrinsic Analysis (AIA) mechanism is used at the second level to convert features into an encoded format that helps mitigate inference attacks. While the second level of privacy is for data transformation and model generation, the first level of privacy focuses on data integrity using blockchain technology for collected observational data (sensor data). The CPS network is protected from inference and poisoning attacks by the transformation of original observational data into a new format using Asymmetric Encryption (AE). A digest of the corresponding record is computed with encryption so that the records’ integrity can be maintained. This message digest produces a one-way cryptographic hash, which is a distinctive signature of fixed length output. Because of the avalanche effect, the one-way cryptographic hash protects against inference and poisoning attacks. This happens because changing only one bit of data can result in a completely different message digest. As a result, data integrity is preserved through the use of this process. In addition, in order to create a block in the BC network, various pieces of information, including the message digest, are combined together to form a block. Any change to a data block causes a chain reaction in the hash, which is simple to verify in CPS networks. Blockchain uses consensus to check the integrity of the hash chain. The traditional consensus method, called Proof-of-Work (PoW), requires a lot of computing power because it requires solving hash puzzles while hash integrity in the network has different levels of difficulty. But, the proposed AILBSM technique is less computationally intensive in terms of proof generation and maintaining the integrity of the hash chain. Then, using estimable Proof-of-Work (ePoW) the message digest will be sent out into the blockchain network. In the LCPoW data authentication algorithm, variables such as number of transactions, previous hash value, block index, timestamp, transactions, proof, and current hash value are taken as inputs for processing, and the block hash value is produced as output. After parameter initialization, blocks are created with appropriate hash values, and if the transaction is valid, the hash value is estimated for the block. If the block index is greater than zero, the digest operation is performed for generating the block hash value. Consequently, the block mining process is executed using ePoW, and proof is returned as output. After that, the new block is added into the blockchain network. In the blockchain, reaching an agreement is a crucial task. When a new record has been verified by enough network nodes, it can be stored in the blockchain. It is not possible to change a block’s contents once it has been verified. Blockchain technology is built in such a way that its validity can be maintained even in the presence of hostile users in untrusted networks. The conventional consensus algorithms require a significant amount of computational power to solve hash puzzles and, varying degrees of difficulty to ensure hash integrity across the network. But, the proposed LCPoW is not computationally complex for proof generation and hash chain integrity.

**Figure:**
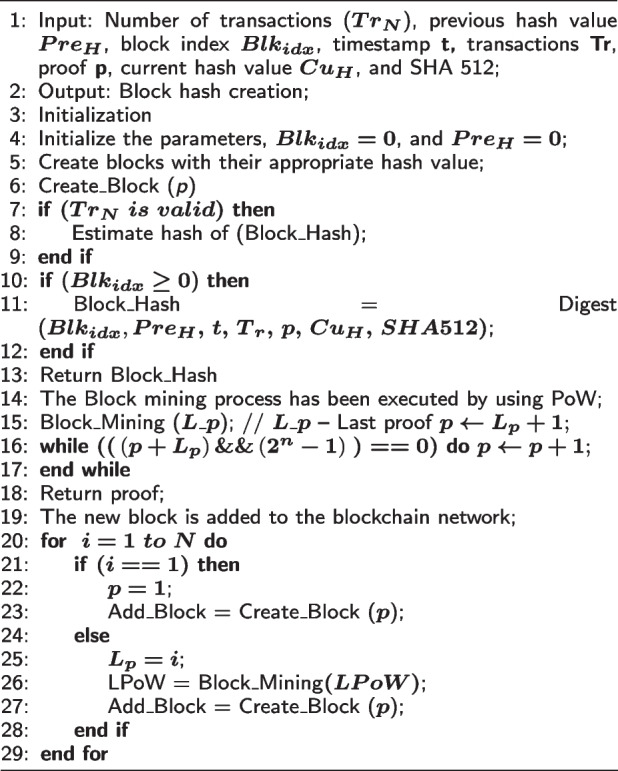
**Algorithm 3** Lightweight Consensus Proof-of-Work (LCPoW) for Data Authentication

### Lightweight Consensus Proof-of-Work (LCPoW) based privacy preservation

The message digest is distributed to the BC network when ePoW is successfully executed, and the raw IIoT sensor data is used for privacy protection. The LCPoW algorithm includes the main operations of block creation, block mining, and insertion of a new block, in which block creation is used to construct the block hash value based on the functions of ${\varvec{Pre}}_{\varvec{H}}$, ${\varvec{Blk}}_{\varvec{idx}}$, $\varvec{t}\varvec{,\ }\varvec{p}\varvec{,\ }\varvec{Tr}$*,* ${\varvec{Cu}}_{\varvec{H}}$, and SHA-512. Typically, the blockchain is one of the most effective solutions to store hash values, and sensor data can be effectively stored in the distributed hash table of the IPFS with its associated hash value. Moreover, this kind of information storage guarantees privacy properties of security, immutability, and authenticity of IIoT systems.

- ***Authentic Intrinsic Analysis (AIA) based Privacy Preservation***

After the data is authenticated, a block is created with the first privacy model and LCPoW is effectively distributed across the blockchain network. Consequently, the second level of privacy protection is provided by using original data of IIoT devices. In the proposed work, an AIA mechanism is used to ensure the second level privacy preservation of the original data obtained from IIoT devices. It consists of attribute mapping, parameter selection, and transformation components.

To this end, this work implements an AIA mechanism that includes attribute mapping, parameter selection, and transformation components. In attribute mapping, the values of categorical variables are transformed into a numerical format to improve efficiency. Then, the best parameters are optimally selected from the given attribute set by eliminating irrelevant features. The main purpose of this selection process is to avoid performance degradation by solving the given problem optimally. This model estimates similarity between two attributes to select the largest possible number of parameters. Here, the correlation coefficient is computed by using the random parameters $\varvec{r}\varvec{1}$ and $\varvec{r}\varvec{2}$ as shown:

1 $$\begin{aligned} {\varvec{Cr}}_{\varvec{e}}\left( {\varvec{r}}_{\varvec{1}}{,\varvec{r}}_{\varvec{2}}\right) \varvec{=}\frac{\int ^{\varvec{x}}_{\varvec{i}\varvec{=}\varvec{1}}{\varvec{(}{\varvec{h}}_{\varvec{i}}\varvec{-}{\overline{\varvec{r}}}_{\varvec{1}}\varvec{)(}{\varvec{q}}_{\varvec{i}}\varvec{-}{\overline{\varvec{r}}}_{\varvec{2}}\varvec{)}}}{\sqrt{\int ^{\varvec{x}}_{\varvec{i}\varvec{=}\varvec{1}}{{\left( {\varvec{h}}_{\varvec{i}}\varvec{-}\overline{{\varvec{r}}_{\varvec{1}}}\right) }^{\varvec{2}}}}\varvec{\ }\sqrt{\int ^{\varvec{x}}_{\varvec{i}\varvec{=}\varvec{1}}{{\left( {\varvec{q}}_{\varvec{i}}\varvec{-}\overline{{\varvec{r}}_{\varvec{2}}}\right) }^{\varvec{2}}}}} \end{aligned}$$

2 $$\begin{aligned} {\overline{\varvec{r}}}_{\varvec{1}}\varvec{=}\left| \frac{\varvec{1}}{\varvec{x}}\int ^{\varvec{x}}_{\varvec{i}\varvec{=}\varvec{1}}{{\varvec{h}}_{\varvec{i}}}\right| \end{aligned}$$

3 $$\begin{aligned} {\overline{\varvec{r}}}_{\varvec{2}}\varvec{=}\left| \frac{\varvec{1}}{\varvec{x}}\int ^{\varvec{x}}_{\varvec{i}\varvec{=}\varvec{1}}{{\varvec{q}}_{\varvec{i}}}\right| \end{aligned}$$

where ${\varvec{Cr}}_{\varvec{e}}\left( {\varvec{r}}_{\varvec{1}}{,\varvec{r}}_{\varvec{2}}\right)$ indicates the correlation coefficient between random integers, as well as  ${\varvec{h}}_{\varvec{i}}$ and ${\varvec{q}}_{\varvec{i}}$ representing the data points for the given attributes. Moreover, it efficiently transforms the parameters into a new dimension without losing more information from the data. Let us consider that the dataset has zero mean values and attributes as shown in:

4 $$\begin{aligned} \varvec{P}\left( \varvec{d}\right) \varvec{=}{\left. \left( {\varvec{k}}_{\varvec{i}}\varvec{-}{\varvec{c}}_{\varvec{i}}\right) \right. }^{\varvec{X}}_{\varvec{i}\varvec{=}\varvec{1}} //\varvec{d}\varvec{=}\varvec{1},\varvec{2}\varvec{\dots }\varvec{s} \end{aligned}$$

where ${\varvec{k}}_{\varvec{i}}$ represents the data sample and ${\varvec{c}}_{\varvec{i}}$ represents the target class. After that, the covariance matrix is formulated by using:

5 $$\begin{aligned} \varvec{\vartheta }\varvec{=}\frac{\varvec{1}}{\varvec{s}\varvec{-}\varvec{1}}\int ^{\varvec{s}}_{\varvec{d}\varvec{=}\varvec{1}}{\varvec{[}\varvec{P}\left( \varvec{d}\right) {\varvec{P}\left( \varvec{d}\right) }^{\varvec{t}}\varvec{]}} \end{aligned}$$

Consequently, the linear transformation $\varvec{\beta }\left( \varvec{d}\right)$ is computed from $\varvec{P}\left( \varvec{d}\right)$ by using:

6 $$\begin{aligned} \varvec{\beta }\left( \varvec{d}\right) \varvec{=}{\varvec{M}}^{\varvec{t}}\varvec{P}\left( \varvec{d}\right) \end{aligned}$$

where ***M*** indicates an orthogonal matrix, and its i${}^{th}$ covariance matrix column *G* is equal to the covariance matrix *i*, which is used to solve the eigen problem based on:

7 $$\begin{aligned} {\varvec{\varepsilon }}_{\varvec{i}}{\varvec{u}}_{\varvec{i}}\varvec{=}{\varvec{Gu}}_{\varvec{i}} \end{aligned}$$

where ${\varvec{\varepsilon }}_{\varvec{i}}$ indicates the eigenvalue, ${\varvec{u}}_{\varvec{i}}$ represents the eigenvector, and the feature components are extracted by using:

8 $$\begin{aligned} {\beta }_i\left( d\right) =u^t_iP\left( d\right) ,\ i=1,2\dots s \end{aligned}$$

Consequently, the projection of the new sample and its associated error values are computed based on:

9 $$\begin{aligned} \hat{P}\left( d\right) =\int ^n_{i=1}{b^t_i(d)b_i} \end{aligned}$$

10 $$\begin{aligned} E_d={Dis}_u(P\left( d\right) ,\hat{P}\left( d\right) ), \end{aligned}$$

where $B=\{b_i:b_i=u_i,i=1,2,\dots n\}$. These mathematical equations are considered as a function used to transform the obtained data into an encoded format, which helps to prevent IIoT systems from dangerous attacks.

### Convivial Optimized Sprinter Neural Network (COSNN)

In this phase, a novel COSNN-based AI method for anomaly detection in IIoT systems is implemented to retrieve input features from the two-level privacy preservation modules for training.

When compared to the classification approaches, the COSNN technique effectively minimizes training time and error rate of anomaly detection. Moreover, COSNN categorizes the attacking instances according to features obtained from the privacy preservation module.

**Fig. 3:**
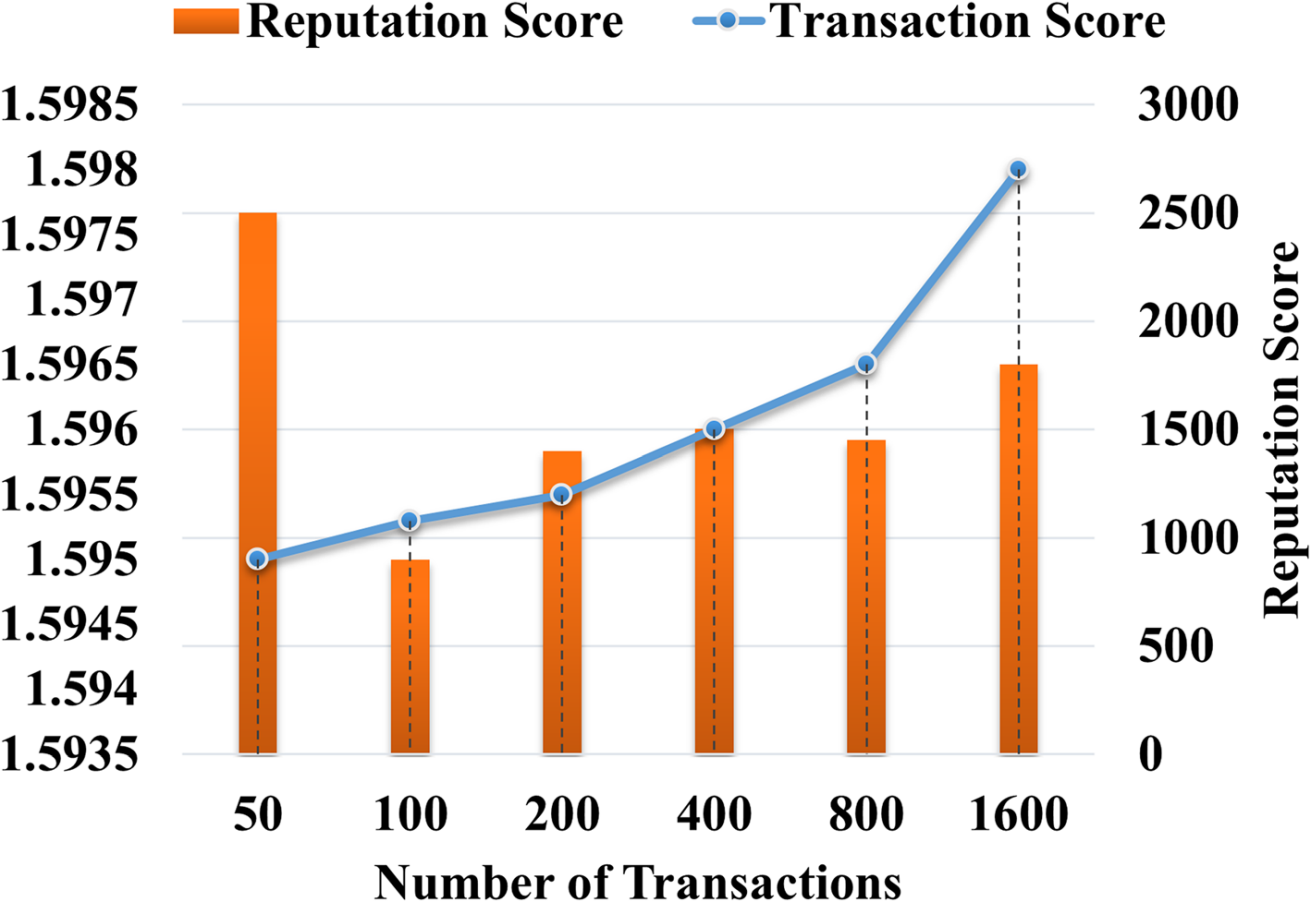
Reputation and transaction score analysis

Here, the most popular and publicly available attack datasets such as NSL-KDD, DS2OS, BoT-IoT, and UNSW-NB15 were used to evaluate the security mechanism that covers all current attacks and can be used for analysis. COSNN is one of the intelligent machine learning classification algorithms that accurately categorizes both normal and abnormal attack classes [40], which includes input, hidden, and output layers. The main advantages of this technique are less processing time, less overfitting and better classification performance. The architectural model of the proposed COSNN mechanism is shown in Fig. 3, where the network is built by linking the outputs of the neurons. The input of this classifier is:

11 $$\begin{aligned} \varvec{D}_{\varvec{S}}\varvec{=}\varvec{\{}{\varvec{D}}^{\varvec{1}}_{\varvec{s}},{\varvec{D}}^{\varvec{2}}_{\varvec{s}}\varvec{\dots }{\varvec{D}}^{\varvec{\delta }}_{\varvec{s}}\varvec{\dots }{\varvec{D}}^{\varvec{M}}_{\varvec{s}}\} \varvec{1}\varvec{\le }\varvec{\delta }\varvec{\le }\varvec{M} \end{aligned}$$

Here, input neurons accept the overall features as inputs, and weight values are used in this allocation for improvising detection processes. Then, the neuron weight value is adjusted in the hidden layer using:

12 $$\begin{aligned} \varvec{\omega }\varvec{=}\varvec{\{}{\varvec{\omega }}_{\varvec{1}},{\varvec{\omega }}_{\varvec{2}}\varvec{\dots }{\varvec{\omega }}_{\varvec{M}}\} \end{aligned}$$

Moreover, the bias function is computed in the hidden layer for producing the output weight value, where ${\varvec{\omega }}^{\varvec{M}\varvec{+}\varvec{2}}$ indicates the weight value of the output neuron, and ${\varvec{\omega }}^{\varvec{M}\varvec{+}\varvec{3}}$ represents the bias function of the output layer. Consequently, the transfer function is computed by using:

13 $$\begin{aligned} T_{s}=\omega ^{M+2} \times \left[ logsig\left( \int _{\delta }^{M} D_{s}^{\delta } \times \omega ^{\delta }+\omega ^{M+1}\right) \right] \nonumber \\ +\omega ^{M+3} \end{aligned}$$

where **logsig** indicates the log sigmoid transfer function that determines the output of the network, $\varvec{\delta }^{th}$ is the neuron input given in ${\varvec{D}}^{\varvec{\delta }}_{\varvec{s}}$,  $\varvec{\ }\varvec{\delta }^{th}$ is the neuron weight given in ${\varvec{\omega }}^{\varvec{\delta }}$, while  ${\varvec{\omega }}^{\varvec{M}\varvec{+}\varvec{1}}$ and ${\varvec{\omega }}^{\varvec{M}\varvec{+}\varvec{3}}$ are the bias values. Based on this function, the output label is produced as normal or attack. The training process of this classifier is improved by using a social optimization algorithm, which is mainly used to increase detection accuracy. This optimization technique includes the following operations: initialization of parameters, estimation of the fitness function, updating position, updating attackers, checking feasibility of the solution and termination. During initialization, the sprinters are randomly initialized according to:

14 $$\begin{aligned} {\varvec{G}}_{\varvec{k}}\varvec{=}\left. {\varvec{G}}_{\varvec{k}}\left( \varvec{x},\varvec{y}\right) \right. \varvec{;}\varvec{1}\varvec{\le }\varvec{x}\varvec{\le }\varvec{M},\varvec{1}\varvec{\le }\varvec{y}\varvec{\le }\varvec{D} , \end{aligned}$$

where **M** indicates the overall stringers, **D** is the dimension of coordinates, ${\varvec{G}}_{\varvec{k}}\left( \varvec{x},\varvec{y}\right)$ indicates the location of stringer *y* at location *x*. Once the group initialization is completed, the input parameters of stringers are consequently initialized, which includes an accelerator ${\varvec{A}}_{\varvec{k}}$, brake ${\varvec{B}}_{\varvec{k}}$, gear ${\varvec{P}}_{\varvec{k}}$, and steering ${\varvec{S}}_{\varvec{k}}$. Then, the fitness function is estimated to find the best feasible solution for identifying intrusions or anomalies, which is computed using:

15 $$\begin{aligned} {\varvec{B}}_{\varvec{F}}\varvec{=}\frac{\varvec{1}}{\varvec{\gamma }}\int ^{\varvec{\gamma }}_{\varvec{s}\varvec{=}\varvec{1}}{\left[ {\varvec{C}}^{\varvec{*}}_{\varvec{s}}\varvec{-}{\varvec{C}}_{\varvec{s}}\right] } \end{aligned}$$

where $\varvec{\gamma }$ represents entire samples, ${\varvec{C}}^{\varvec{*}}_{\varvec{s}}$ is the targeted output, and ${\varvec{C}}_{\varvec{s}}$ represents the categorized output. Then, the leading position of the string is identified based on the fitness function, and the position of the string is updated based on the identification of a winner. Moreover, the location can be further changed based on the properties of stringers, and positional update is performed using:

16 $$\begin{aligned} {\varvec{G}}^{\varvec{a}}_{\varvec{k}\varvec{+}\varvec{1}}\left( \varvec{x},\varvec{y}\right) \varvec{=}\varvec{\alpha }[{\varvec{G}}_{\varvec{k}}\left( \varvec{\vartheta },\varvec{y}\right) \varvec{\times }\varvec{\varphi }\left( \varvec{y}\right) \varvec{+}\nonumber \\ {\varvec{D}}_{\varvec{s}}\varvec{(}\varvec{\delta },\varvec{y}\varvec{)\times (}\varvec{1}\varvec{-}\varvec{\varphi }\varvec{(}\varvec{y}\varvec{))}] \end{aligned}$$

where $\varvec{\varphi }\varvec{=}\varvec{x}$, $\varvec{\alpha }$ indicates the random integer in the range of 0 to 1, $\varvec{\vartheta }$ is the random number, and $\varvec{\varphi }$ is also random with a range from 0 to 1, then the location of every individual is updated. Moreover, the location of followers and overtakers are updated according to direction, travelling distance, angle, and relative success rate. In addition, the attacker’s location update is performed with the update of the leading stringer using:

17 $$\begin{aligned} {\varvec{G}}^{\varvec{v}}_{\varvec{k}\varvec{+}\varvec{1}}\left( \varvec{x},\varvec{y}\right) \varvec{=}{\varvec{G}}^{\varvec{H}}\left( \varvec{H},\varvec{y}\right) \varvec{+}{\varvec{\textrm{cos}} \left( {\varvec{K}}^{\varvec{k}}_{\varvec{x},\varvec{y}}\right) \ }\varvec{\times }\nonumber \\ {\varvec{G}}^{\varvec{H}}\left( \varvec{H},\varvec{y}\right) \varvec{+}{\varvec{e}}^{\varvec{k}}_{\varvec{x}} \end{aligned}$$

where ${\varvec{G}}^{\varvec{H}}$ indicates the leading position of the stringer, ${\varvec{K}}^{\varvec{k}}_{\varvec{x},\varvec{y}}$ is the stringer’s steering angle at coordinate *y*, and ${\varvec{e}}^{\varvec{k}}_{\varvec{x}}$ represents the distance travelled by the *x*${}^{th}$ driver. Furthermore, the best stringer is selected based on the best fitness value, and the parameters of the selected stringer are optimally updated, including gear and steering angle. Finally, the termination condition is executed once the maximum number of iterations is reached. Through this optimization, the overall performance of the proposed COSNN classification technique in detecting attacks is greatly improved.

## Results and discussions

In this section, we present the experimental analysis of the proposed AILBSM framework using different evaluation indicators. Here, the performance of both the blockchain-based privacy preservation and the COSNN-based AI mechanisms were validated and tested using execution time, trust score, Precision, Recall, Accuracy, and F1 score. Figure 2 validates the execution time of the LCPoW blockchain model with respect to the different number of IIoT devices in the network. Here, the execution time for securely uploading the different file types from sensor devices to the IPFS system is estimated. Our in-depth analysis shows that the proposed privacy preservation model requires minimum execution time for storing information with high security. Similarly, the reputation and transaction values for the proposed privacy preservation model are estimated with respect to different number of transactions in IIoT systems. In the proposed framework, each sensor device is assigned a unique address in the blockchain network, and the reputation value of each sensor is estimated based on transaction value, as shown in Fig. 3.

**Fig. 4:**
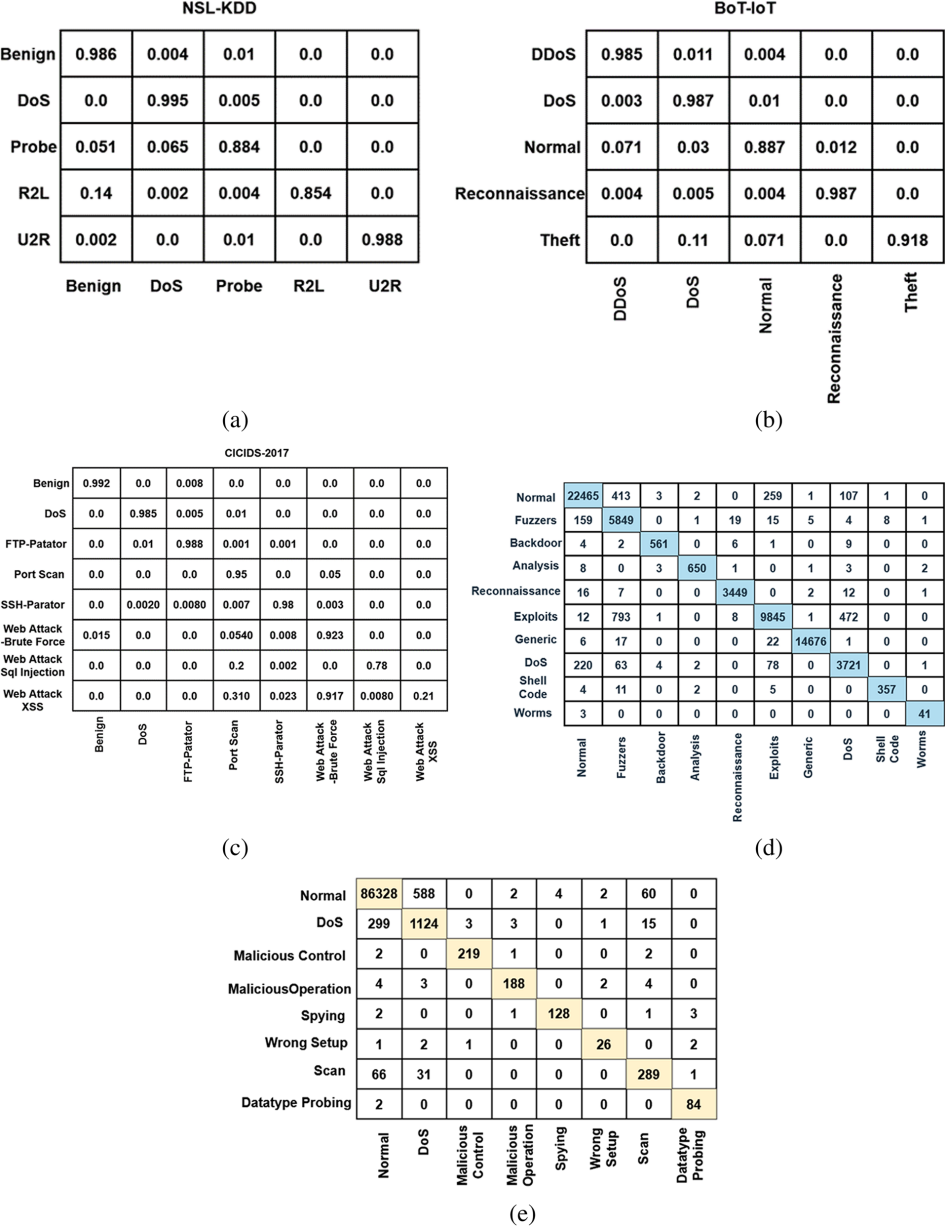
Confusion matrix **a)** NSL-KDD dataset, **b)** BoT-IoT IDS, **c)** CICIDS 2017, **d)** UNSW-NB 15, and **e)** DS2OS dataset

Figure 4 (a) to (e) present the generated confusion matrix of the proposed COSNN-based AI mechanism for different types of datasets. Typically, the confusion matrix is mainly used to validate the detection performance of the classifier. According to the improved True Positive Rate (TPR), the classifier’s increased accuracy is determined. In this analysis, the confusion matrices are validated for all types of cyber threat datasets. Our positive results prove that the combination of the proposed COSNN mechanism provides accurate predicted results by correctly detecting intrusions and their appropriate classes.

**Fig. 5:**
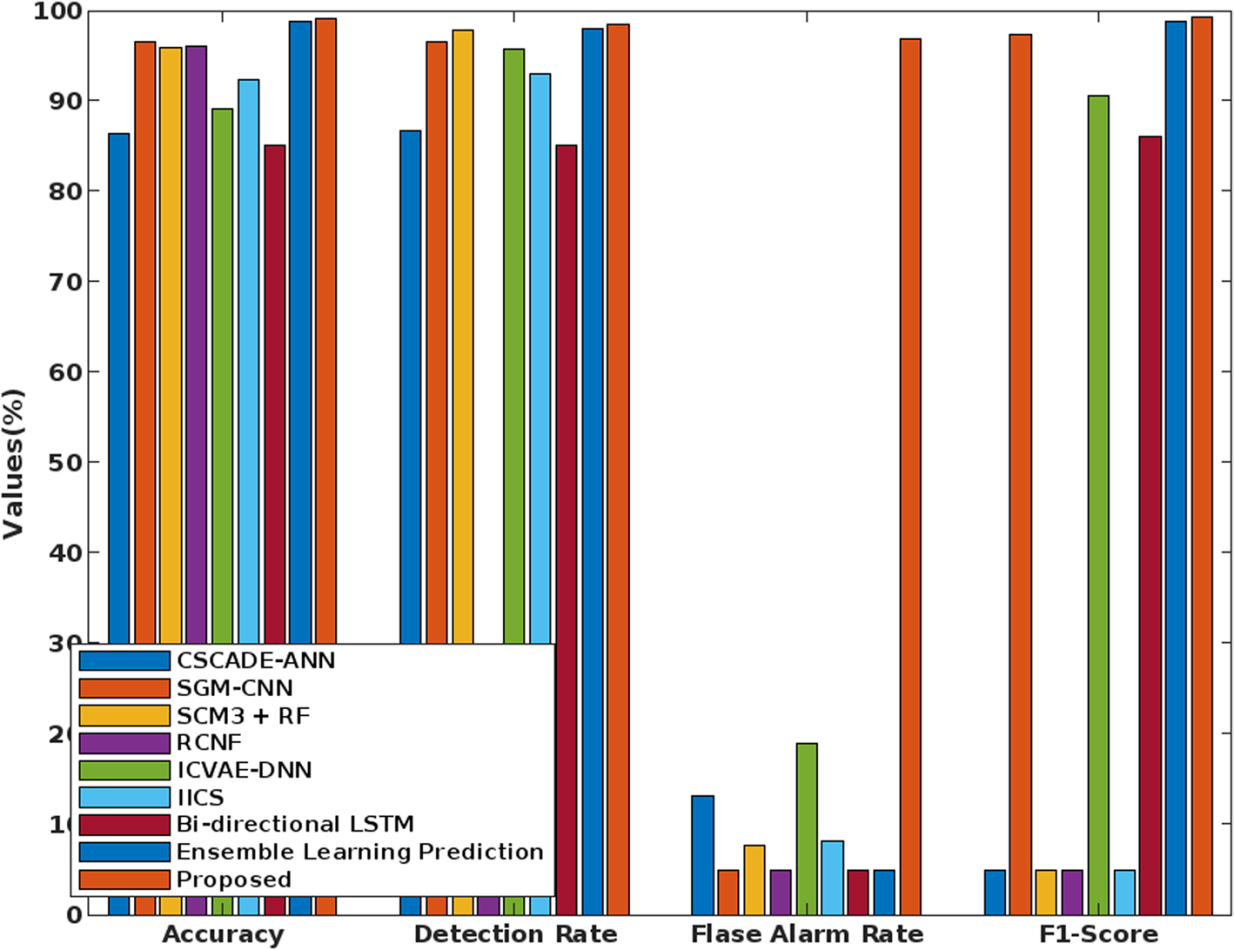
Overall performance analysis

Figure 5 presents the overall performance analysis of the conventional and the proposed classification-based approaches for intrusion detection. It includes known techniques like Semi Global Matching - Convolutional Neural Network (SGM-CNN), Real Time Collaborative Network (RCNF), SGM3 - Random Forest (RF), Improved Conditional Variational Auto Encode (ICVAE) - Deep Neural Network (DNN), Internet Industrial Control System (IICS), and Cascaded Artificial Neural Network (CSCADE-ANN).

**Fig. 6:**
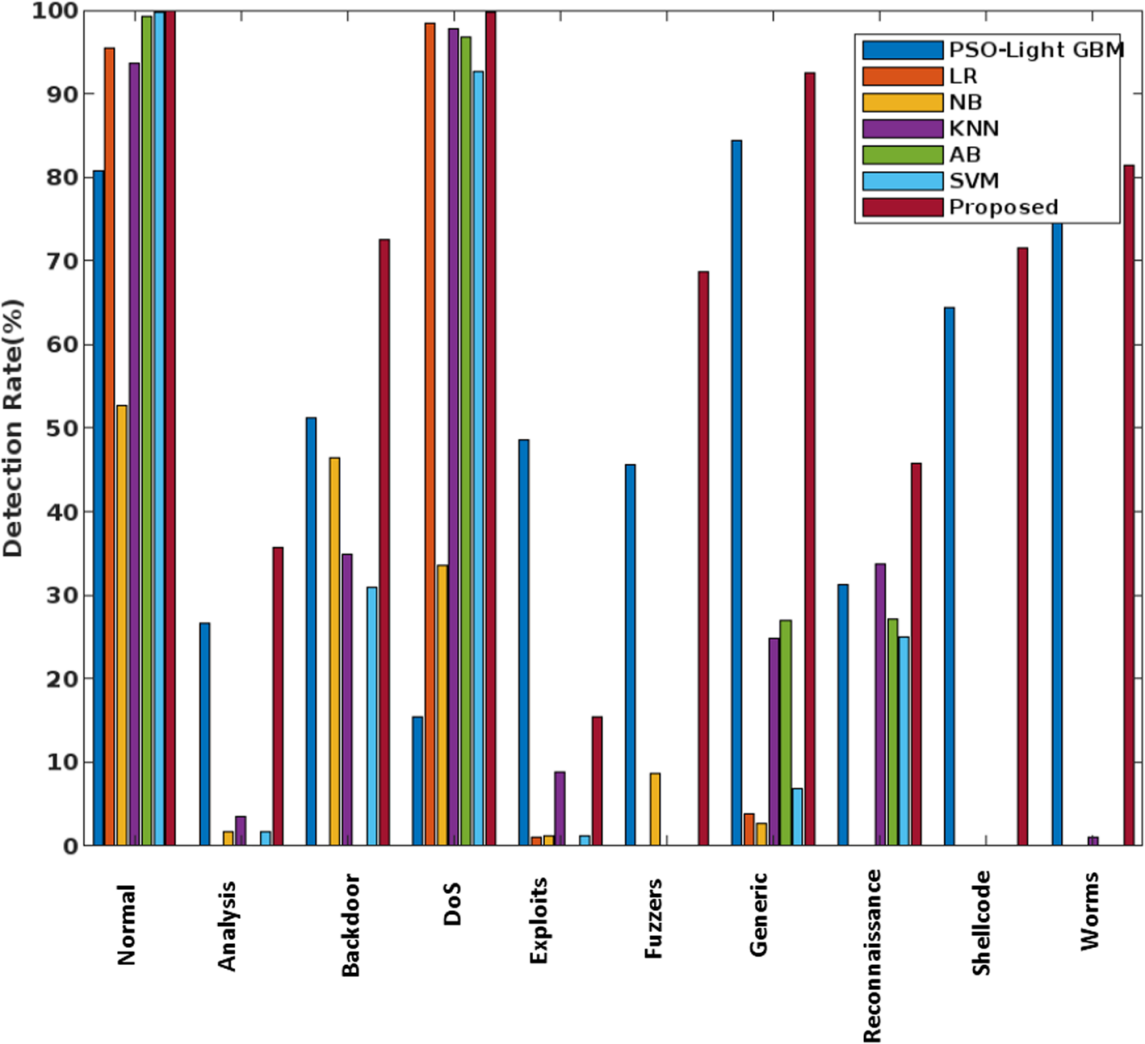
The detection rate of various machine learning techniques using the UNSW-NB 15 dataset

Here, the results are evaluated in terms of Accuracy, detection rate, False Alarm Rate (FAR), and F1 score. From the results, the proposed COSNN technique outperforms other approaches with better performance results. Consequently, the detection rate for the state of the art IDS [28] is validated as shown in Fig. 6. In this evaluation, the detection rate is evaluated for nine attacker classes and one normal class of the UNSW-NB15 dataset. Among other mechanisms, the proposed COSNN technique has an excellent detection rate for most attack classes, especially worms, shellcodes, and generic cases. Moreover, the proposed technique is very robust and reliable, and therefore has strong detection performance compared to other classification approaches.

**Fig. 7:**
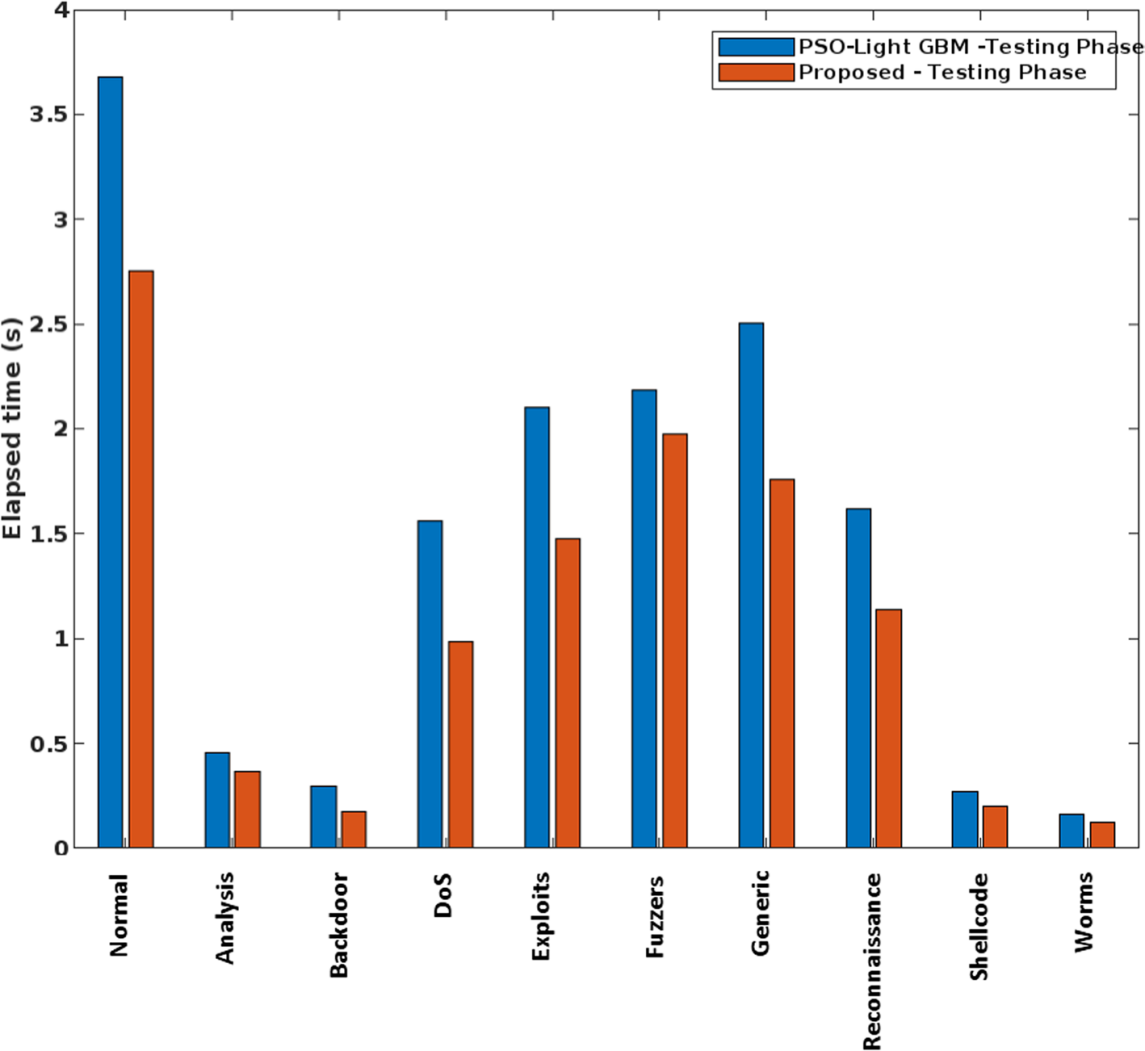
Elapsed time analysis using UNSW-NB 15 dataset

**Fig. 8:**
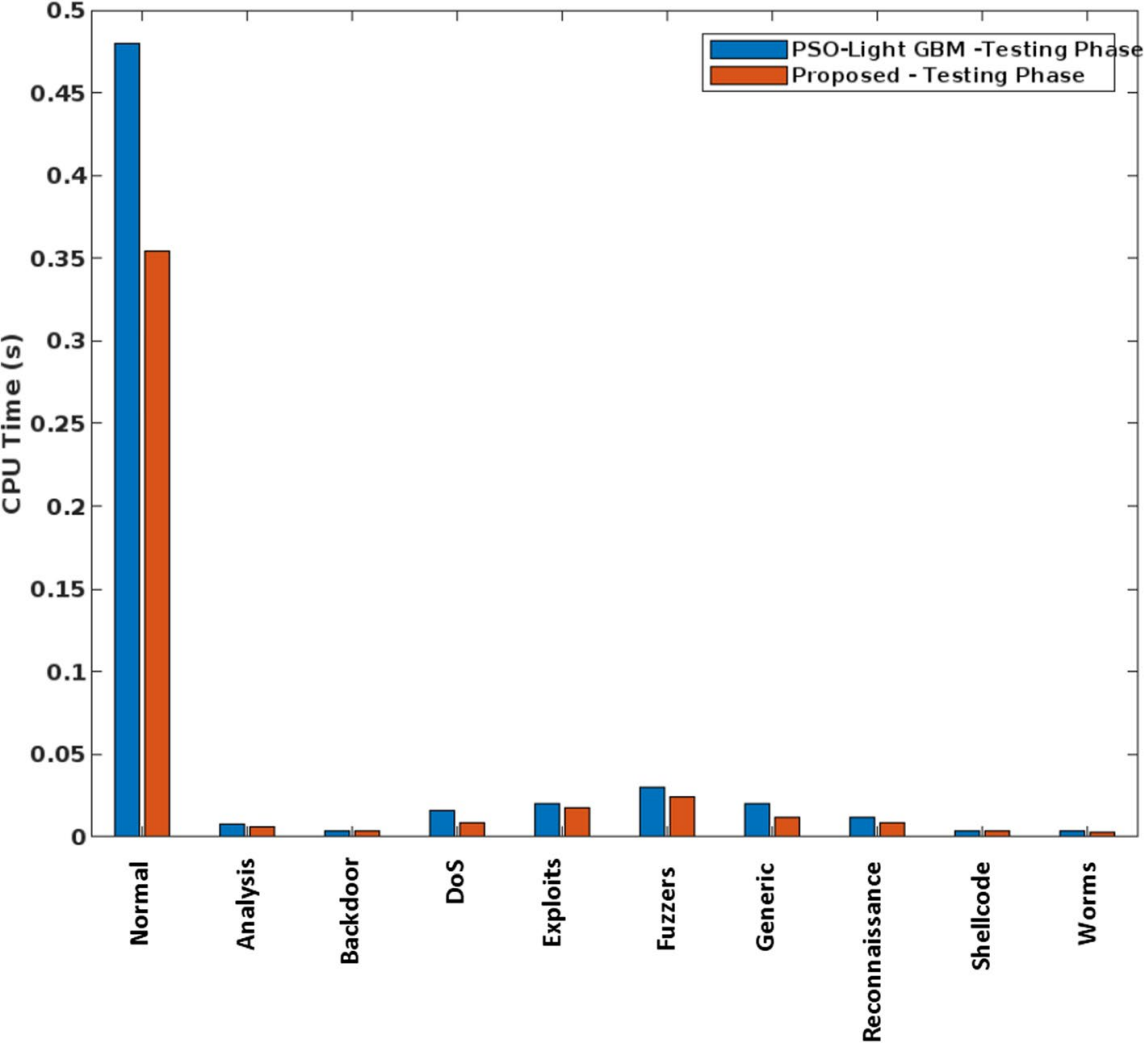
CPU time analysis using UNSW-NB 15 dataset

**Fig. 9:**
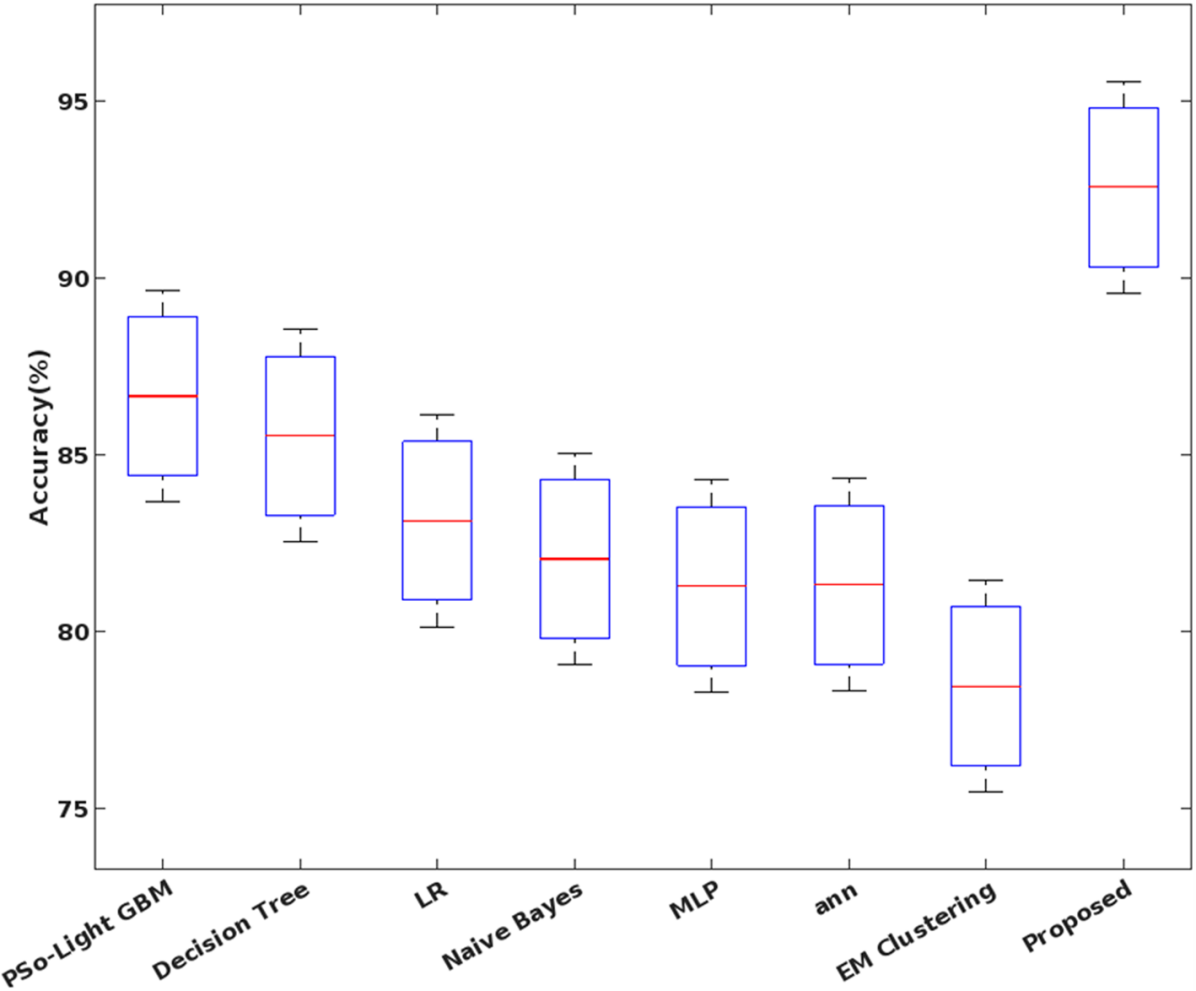
Accuracy of machine learning classifiers using UNSW-NB dataset

In addition, the elapsed time and the CPU execution time of conventional and the proposed safety approach are validated and compared in Figs. 7 and 8, respectively. Here, the time analysis is performed according to different attack classes in the UNSW-NB15 dataset. Typically, the time cost of training and testing a classifier can vary greatly in proportion to the type of predicted classes. For example, a typical class has the largest proportion during training and testing and therefore requires more time with a low frequency of data. The observed results show that the proposed COSNN technique requires less time compared to conventional approaches. Moreover, the Accuracy of standard machine learning models and the proposed classification model is validated using the UNSW-NB15 dataset, as shown in Fig. 9.

**Fig. 10:**
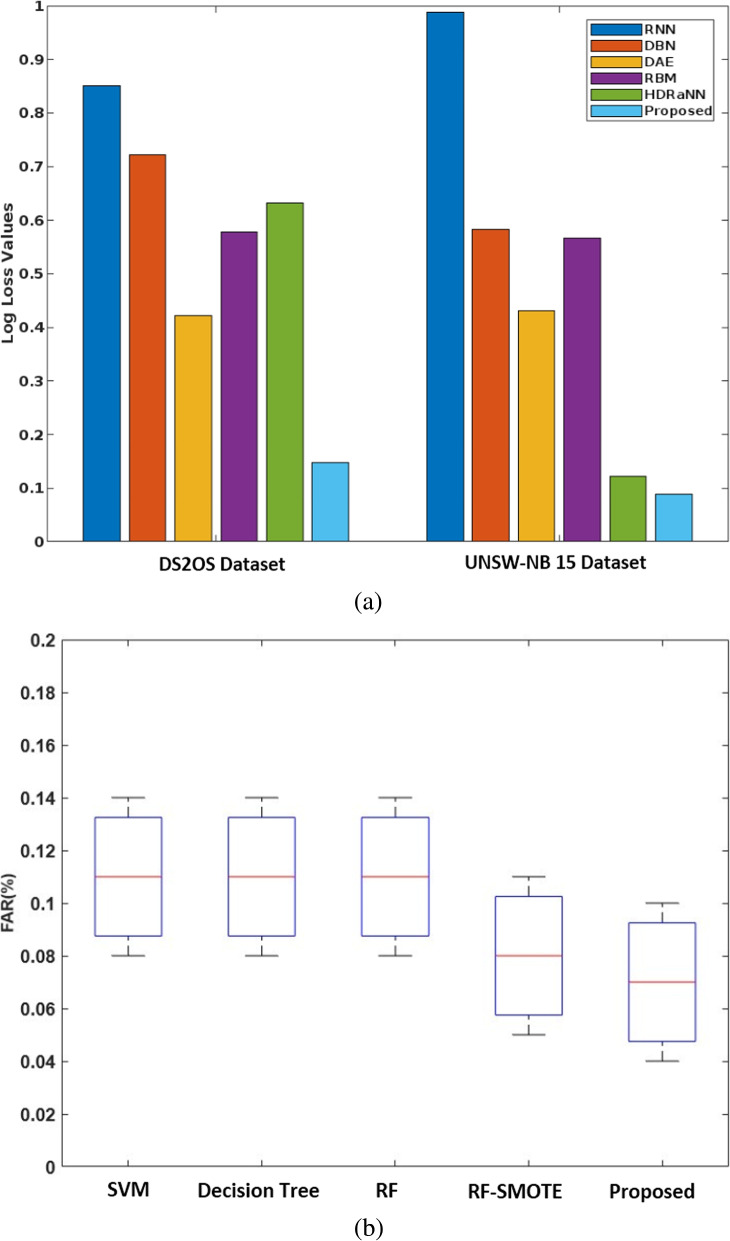
**a** Log loss value and **b** FAR of BoT-IoT IDS dataset

Figure 10 (a) validates the log-loss value of existing and proposed classification techniques for two datasets, DS2OS and UNSW-NB15, respectively. Normally, the log-loss value should be minimized to ensure accurate detection, since an increased loss value may affect the performance of the overall security model. Based on our analysis, the proposed COSM-RMML technique reduces the log-loss value for both analyzed datasets by adequately processing the input datasets. Moreover, the False Acceptance Rate (FAR) of standard machine learning algorithms versus the proposed techniques are validated and compared using the BoT-IoT IDS dataset, as shown in Fig. 10 (b). By properly training and testing the features in the classifier, the FAR of the proposed classifier is effectively reduced compared to other approaches.

## Conclusion

This paper presents a novel, hybrid blockchain-integrated AI-based security framework coined as the  Artificial Intelligence-based Lightweight Blockchain Security Model (AILBSM) to guarantee security and privacy of IIoT systems. Here, the BlockCloud (BC) and BlockFog (BF) platforms are utilized to solve security challenges in standard cloud-fog systems. The proposed framework is make up of two modules of security operations, privacy preservation and anomaly detection respectively. During privacy preservation, reputation-based trust estimation, LCPoW, and AIA mechanisms are utilized for secured data storage in the BC and BF systems. This model treats the sensor device with an increased reputation score as the trusted device. The normal, good, and malicious transactions are categorized in the IIoT systems based on the trust score. If the identified transaction is valid, the information collected from the IIoT sensors is correctly stored in the IPFS, ensuring security and privacy properties.

**Fig. 11:**
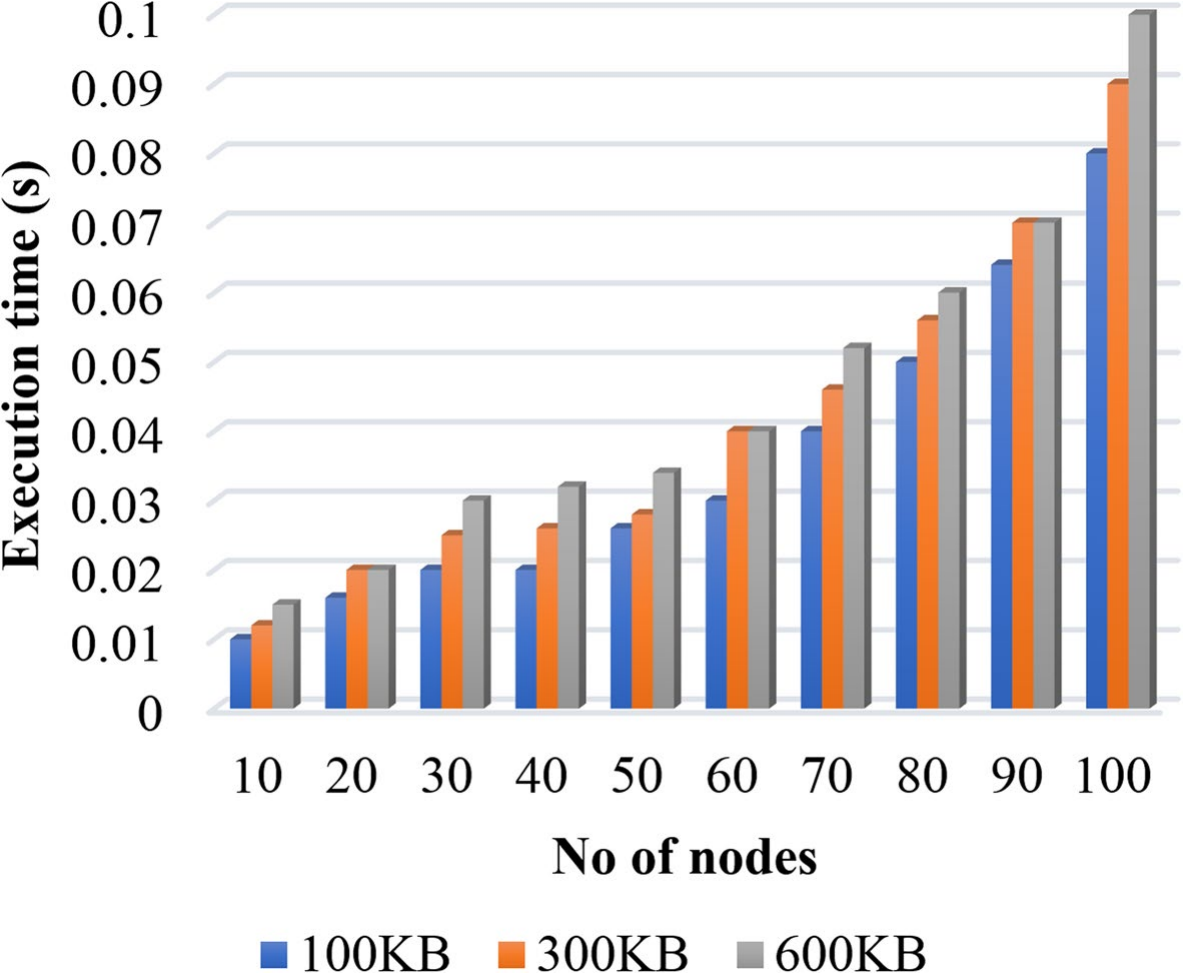
Execution time analysis

In Algorithm 3, we provide an Lightweight Consensus Proof-of-Work (LCPoW) strategy that is less computationally expensive in terms of proof production and upholding hash chain integrity, when compared to the other consensus algorithms. The message digest is distributed to the blockchain network after the LCPoW has been successfully executed, and actual data produced by the Industrial Internet of Things (IIoT) sensors is then protected using second level privacy. This digest reduces the likelihood of an attack. On the BlockFog node, the generated hash is distributed for the digest’s verification. The data is transmitted to an Artificial Intelligence (AI) model for a second level of privacy after it has been successfully authenticated and added to a block in the blockchain network. The observational data (raw data) is transformed into a new format by the underlying privacy mechanism. Moreover, the performance of this mechanism is validated and assessed in terms of time consumption as shown in Fig. 11. The observed results indicate that the execution time is effectively reduced with the use of the LCPoW algorithm.

 The LCPoW algorithm is implemented to authenticate data to prevent IIoT systems from harmful attacks, which is made up of significant operations of block creation, mining, and insertion of new blocks. Consequently, an Authentic Intrinsic Analysis (AIA) mechanism is deployed in the second level to transform the features into an encoded format that helps mitigate attack inference, including components like attribute mapping, parameter selection, and transformation. Finally, a novel COSNN-based AI methodology is implemented to detect the anomalies in the IIoT systems, which obtains the input features from the two-level privacy preservation modules for training. During the evaluation, the performance of both blockchain-based privacy preservation and the Convivial Optimized Sprinter Neural Network (COSNN)-based AI mechanisms was validated and tested using execution time, trust score, Precision, Recall, Accuracy, and F1 score. In the AILBSM framework, the execution time is reduced to 0.6 seconds, the overall classification accuracy is improved to 99.8%, and the detection performance is increased to 99.7%, respectively. When compared to other approaches, the anomaly detection performance of the proposed model has been significantly enhanced due to the incorporation of auto-encoder-based transformation and blockchain authentication. Overall, the results indicate that the proposed AILBSM framework provides improved results with high computational efficiency, reduced time consumption, and high security.

## Data Availability

Not applicable.
